# Current challenges in secondary progressive multiple sclerosis: diagnosis, activity detection and treatment

**DOI:** 10.3389/fimmu.2025.1543649

**Published:** 2025-03-21

**Authors:** Luis Brieva, Carmen Calles, Lamberto Landete, Celia Oreja-Guevara

**Affiliations:** ^1^ Neurology Department, Hospital Universitari Arnau de Vilanova, Lleida, Spain; ^2^ Medicine Department, Universitat de Lleida (UdL), Lleida, Spain; ^3^ Neuroimmunology Group, Institut de Recerca Biomedica de Lleida (IRBLLEIDA), Lleida, Spain; ^4^ Neurology Department, Hospital Universitario Son Espases, Palma de Mallorca, Spain; ^5^ Neurology Department, Hospital Universitario Doctor Peset, Valencia, Spain; ^6^ Department of Neurology, Hospital Clinico San Carlos, Instituto de Investigación Sanitaria del Hospital Clínico San Carlos (IdISSC), Madrid, Spain; ^7^ Departament of Medicine, Medicine Faculty, Universidad Complutense de Madrid (UCM), Madrid, Spain

**Keywords:** multiple sclerosis, secondary progressive multiple sclerosis, disease activity, silent progression, smouldering disease, multiple sclerosis treatment, disease-modifying treatments

## Abstract

Approximately 50% diagnosed with relapsing-remitting multiple sclerosis (RRMS) transition to secondary progressive multiple sclerosis (SPMS) within 20 years following disease onset. However, early diagnosis of SPMS and effective treatment remain important clinical challenges. The lack of established diagnostic criteria often leads to delays in identifying SPMS. Also, there are limited disease-modifying therapies (DMTs) available for progressive forms of MS, and these therapies require evidence of disease activity to be initiated. This review examines the challenges in diagnosing SPMS at an early stage and summarizes the current and potential use of biomarkers of disease progression in clinical practice. We also discuss the difficulties in initiating the DMTs indicated for active SPMS (aSPMS), particularly in patients already undergoing treatment with DMTs that suppress disease activity, which may mask the presence of inflammatory activity required for the therapy switch. The article also addresses the DMTs available for both active and non-active SPMS, along with the clinical trials that supported the approval of DMTs indicated for aSPMS or relapsing MS in Europe, which includes aSPMS. We also offer insights on when discontinuing these treatments may be appropriate.

## 1 Introduction

Multiple Sclerosis (MS) is a chronic autoimmune disorder of the central nervous system (CNS), characterized by neuroinflammation, demyelination, and neurodegeneration. Approximately, 2.9 million individuals are affected by MS worldwide, with the average age at diagnosis being 32 years (1). MS places a huge burden on both individuals and society as a whole (2). The impact of the disease on daily living, work-related activities, and cost becomes more pronounced with the progression of disability (3, 4).

The clinical manifestations and course of MS are unpredictable and vary from person to person. Standardized definitions of MS clinical courses are important for clinicians to diagnose, monitor, and treat their patients, as well as for researchers to design studies, and interpret and compare findings. MS was initially classified into four phenotypes: relapsing-remitting MS (RRMS), primary progressive MS (PPMS), secondary progressive MS (SPMS), and progressive-relapsing MS (PRMS) (5). In the revision of 2013 by the International Advisory Committee on Clinical Trials of MS (6), patients were classified according to the presence of activity (active or non-active) and disease progression (progressing or non-progressing). These phenotypes described in 2013 are still not widely used by the entire scientific community. Moreover, the classification of MS varies between different organizations (the Food and Drug Administration [FDA], the European Medicines Agency [EMA], and the MS scientific community), which can lead to discrepancies in diagnosis and treatment (7). These phenotypes, in any case, seem to represent different stages along the MS disease continuum rather than distinct disease categories (8, 9).

In natural history studies examining untreated cohorts, about 50% of patients progressed to SPMS within 10 to 20 years following disease onset (5, 10, 11). The introduction of disease-modifying therapies (DMTs) three decades ago significantly delayed this conversion rate (12, 13). Findings from DMT-treated cohorts showed that 18% of patients progressed to SPMS after a median duration of 16.8 years from MS onset (13), which is an important reduction compared to untreated cohorts. Factors increasing the risk of conversion from RRMS to SPMS include older age at onset, smoking, higher Expanded Disability Status Scale (EDSS) at onset, a high number of early relapses, motor dysfunction, cerebellar dysfunction, and presence of lesions in the spinal cord (10, 11). From an EDSS score of 3, patients seem to progress at a similar rate, independent of their prior disease course. A study with 2054 patients (1609 relapsing/445 progressive onset) showed that disability progression in the period until irreversible EDSS 3 did not influence disability progression during the period from irreversible EDSS 3 to irreversible EDSS 6 (14).

Despite these increases in the delay of progression due to DMTs, there is an underestimation of the size of the SPMS population in certain regions (15), with the prevalence of SPMS widely varying among countries (15, 16). A study involving patients from Germany, the UK, and Sweden demonstrated that the use of a classifier, which allowed for the reclassification of RRMS/SPMS patients, increased the number of patients classified as SPMS, resulting in a more uniform prevalence of SPMS across these countries (15). Potential factors influencing SPMS prevalence include demographic and geographic factors, healthcare resources, operational definitions of SPMS, or reimbursement criteria for DMTs (16, 17).

Notably, the diagnosis of SPMS is usually made retrospectively through clinical assessment, often based on the observation of irreversible disability progression as measured by the EDSS. The delay between the detection of initial signs suggestive of progression and the confirmed diagnosis of SPMS can last up to three years (18). Despite the publication of various national consensus statements (19, 20), unified criteria for defining the onset of SPMS are still lacking. Timely SPMS diagnosis is key because it provides a window for therapeutic intervention. Early signs of MS activity, such as cognitive decline, brain atrophy, and fatigue, are often missed in routine monitoring, which usually focuses on relapses and MRI findings. Silent progression, undetected by these standard methods, contributes to disease worsening. This highlights the need to update MS management strategies, ensuring timely initiation and escalation of DMT (21).

In Europe, five DMTs are approved for relapsing MS (RMS; interferon-beta-a [IFN-β-1a], cladribine, ocrelizumab, ofatumumab, and ponesimod), which includes both RRMS and SPMS with disease activity, one is approved for RRMS and active SPMS (aSPMS; INF-β-b), and another is approved for aSPMS (siponimod). Detecting disease activity in SPMS is difficult not only due to a decrease in inflammatory activity, but also because these patients are often already under treatment with a DMT that effectively controls clinical and radiological activity. This scenario highlights the complexity of SPMS management: the need to identify disease activity to select the appropriate DMT in patients with ongoing treatments that reduces or eliminate such activity.

A narrative review was conducted for the elaboration of the article. No structured method for searching or evaluating the research literature were used. Instead, authors reviewed publications on predefined topics (diagnostic challenges in SPMS, difficulty in detecting disease activity in treated patients and disease-modifying treatments) and then shared the most relevant information of these publications and their opinions on the gaps in current knowledge, current challenges, and future directions in two structured meetings held on February 27, 2024, and April 10, 2024. In these meetings, the discussion was guided by a moderator who also summarized the contributions by the authors and shared them via email following each meeting for final review. This article aims to provide a comprehensive review and position statement on the current challenges associated with SPMS, addressing key issues such as diagnosis, the assessment of disease activity in patients undergoing DMT treatment, and the available therapeutic options for managing aSPMS.

## 2 Diagnostic challenges in SPMS

### 2.1 Diagnostic criteria

The diagnosis of SPMS is still challenging due to the lack of established clinical, imaging, immunological, or pathological criteria that clearly define this progression. Currently, the definition of SPMS by Lorscheider et al. published in 2016 is the most widely used to identify SPMS (22). This definition includes a disability progression by 1 EDSS step in patients with EDSS ≤5.5 or 0.5 EDSS steps in patients with EDSS ≥6 in the absence of a relapse, a minimum EDSS score of 4.0 and Pyramidal Functional System score of 2 and confirmed progression over at least 3 months (22). However, this definition does not capture all patients transitioning to SPMS, as some may experience a progressive course without reaching the specified EDSS threshold. In fact, some national consensus supports recognition of progression without setting a specific minimum EDSS score (19), whereas others advocate for the minimum EDSS score of 4.0 as a criterion for identifying disease progression in MS (20).

In clinical trials assessing DMTs for SPMS, different inclusion criteria have been established to select participants. In the EXPAND trial (siponimod vs placebo), patients with an EDSS score ranging from 3.0 to 6.5, who had also shown EDSS progression within the two years before the study, were included (23). Similarly, the ASCEND trial (natalizumab vs placebo) required participants to have an EDSS score between 3.0 and 6.5, but also a Multiple Sclerosis Severity Score (MSSS) of 4 or higher, and disability progression independent of relapse activity (PIRA) in the year before the study (24). In the HERCULES trial, which has evaluated the efficacy and safety of tolebrutinib compared with placebo in patients with non-relapsing SPMS (nrSPMS) (25), patients were selected if they had an EDSS from 3.0 to 6.5, had disability progression within the year before the study, and had an absence of clinical relapses for at least two years before the study. The variation in criteria among national consensus and clinical trials exemplifies the ongoing lack of standardization for defining disease progression in MS.

Although the EDSS remains the primary assessment for defining SPMS, this tool is not exempt from limitations (26). In fact, the EDSS mainly focuses on ambulation and physical disability, with cognition, vision, and upper limb function being underrepresented (21). To conduct a complete assessment of disability, the EDSS could be used with other functional assessments, such as the 9-Hole Peg Test (9HPT) and the Timed 25-Foot Walk (T25FW) (20). The Multiple Sclerosis Functional Composite (MSFC) combines the 9HPT, T25FW, and cognition (Paced Auditory Serial Addition Test; PASAT) into one score, providing a multidimensional tool for evaluating functional changes and disease progression. Cognition, which is impaired in most patients with SPMS (27), should be assessed with validated neuropsychological tests. Cognitive assessment to detect progression should be conducted with a neuropsychological evaluation. In those cases in which the administration of a full battery of neuropsychological tests is not possible, applying at least a short and validated test, such as the Symbol Digit Modalities Test (SDMT), for the screening of cognitive function is recommended (20). Electronic self-administered tools and other digital tools can be also practical to assess mobility and cognitive changes. Consistent criteria for identifying cognitive impairment in MS are also needed to improve diagnosis and monitoring disease progression (21).

### 2.2 Pathophysiology

The pathological mechanisms underlying disease progression in MS are complex and not fully elucidated. Both inflammation and neurodegeneration are already present at disease onset (28–32). These pathological processes coexist, to varying degrees and in different CNS locations, throughout the disease course (33, 34). The disease progression seems to involve quantitative changes in the extent of these processes rather than qualitative changes in the type of pathological activity (35, 36). Indeed, no qualitative differences are found when comparing patients with PPMS and SPMS, although there are some quantitative differences. For instance, the presence of focal and active classical white matter lesions and the global degree of inflammation are lower in PPMS compared to SPMS (37). This suggests that RRMS, PPMS and SPMS are part of a disease spectrum modulated by genetics and environmental factors (38).

**Figure 1:**
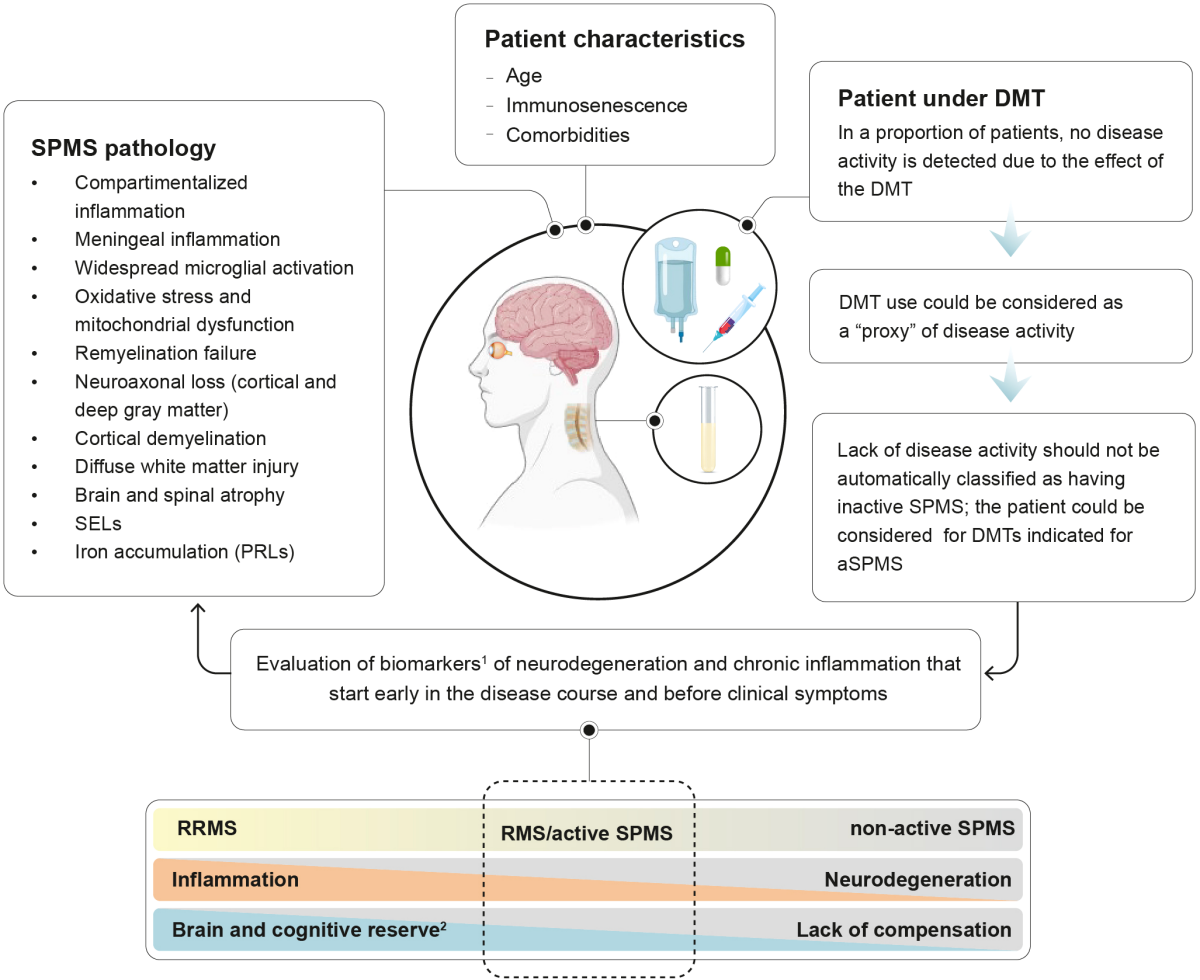
Detecting disease activity in treated patients transitioning to SPMS. ^1^biomarkers of MS progression are presented on **Table 1**; ^2^Brain reserve: structural characteristics of the brain that enable to maintain cognitive function despite brain pathology; cognitive reserve: ability to optimize performance through the differential recruitment of brain networks and alternative cognitive strategies.

The widespread neuroaxonal injury observed in progressive MS seems to be caused by the interplay of several pathological mechanisms, including compartmentalized neuroinflammation within the CNS, axonal degeneration, microglial activation, astrogliosis, oxidative stress, mitochondrial dysfunction, iron toxicity, and deficient remyelination, among others (9, 36, 39, 40). The activation of these mechanisms marks the biological onset of the disease and initiates the prodromal period (**Figure 1**). The dysregulation of the immune system that triggers these pathological mechanisms in the progression of MS includes chronic and elevated levels of pro-inflammatory cytokines (interleukin-1β, interleukin-6, tumor necrosis factor-alpha, interleukin-17, and tumor necrosis factor-alpha), chemokines, and autoantibodies (41).

Additionally, the biological process of aging superimposes the pathogenesis of progressive MS. Immunosenescence, the gradual deterioration of the immune system associated with aging, drives chronic low-grade inflammation, known as inflammaging, which is superimposed over the pathogenesis of MS (42, 43). Moreover, biological aging of glial cells has been observed to be accelerated in the brains of people with MS (44).

The balance of such mechanisms, together with repair capacity and brain and cognitive reserve influences clinical progression during the disease course. Importantly, aging is associated with a decline in brain reserve and cognitive reserve, rendering the CNS less resilient to the cumulative effects of MS pathology (45). Indeed, it has been hypothesized that loss of neurologic reserve explains the onset of progressive MS (46). In the early stages of MS, inflammation leads to brain atrophy, but symptoms are mitigated by the neurologic reserve and functional reorganization of neural networks (47). Over time, as injury from both MS and aging accumulates, the neurologic reserve and the ability to compensate diminishes. This depletion reveals the effects of subclinical MS activity and aging, presenting as progressive MS (46). Therefore, the interplay between age-related neurodegeneration, MS-specific pathological processes, and neurologic reserve explains the progression of disability observed in the advanced stages of the disease. Once the accumulation of irreversible damage surpasses the CNS ability to compensate, disability worsening emerges.

Genetics, environmental and lifestyle factors, including obesity, dysbiosis of the gut microbiota, lack of physical activity, smoking and vitamin D deficiency, play and important role in the progression of MS pathophysiology and the CNS ability to compensate (48–50).

Disability accumulation can result from relapse-associated worsening (RAW) or PIRA (progression independent of relapse activity, or the so-called “silent progression”). Studies with large patient cohorts have demonstrated that PIRA is the main driver of disability accumulation, particularly as the disease advances (29, 51–53). The occurrence of PIRA following an initial demyelinating event is a negative prognostic indicator of the disease course (53). However, the definition of PIRA is mainly based on motor worsening, assessed by the EDSS or EDSS-plus, and does not capture the worsening of other symptoms (independent of relapses), such as subtle motor impairment, cognitive slowing, early fatigability, neuropathic pain, and bowel/bladder and sexual dysfunction (39). The term smoldering-associated worsening (SAW) has been suggested to capture the pathobiological processes associated with these clinical manifestations (35, 39).

### 2.3 Phenotyping MS

Considering the diverse pathological processes underlying MS, several MS phenotyping methods more rooted in the biological mechanisms of MS have been proposed to provide a more comprehensive definition of MS phenotypes than the traditional classification (54–56). For instance, Pitt et al. (54) suggested extending the current classification of MS by including additional pathological processes, such as chronic perilesional inflammation, neuroaxonal degeneration, and remyelination, to improve the phenotyping of MS. Incorporating these processes could help differentiate MS phenotypes that appear clinically similar but are driven by different underlying pathological patterns (54).

MS subtypes based on pathological features using MRI have also been proposed (55). The three subtypes identified in one study using unsupervised machine learning were cortex-led, normal-appearing white matter-led, and lesion-led. Patients with the lesion-led subtype had the highest risk of confirmed disability progression (CDP) and relapse and showed positive DMT response in selected clinical trials (55).

Immune signatures in the blood have also been used as a surrogate of disease pathophysiology to explain MS endophenotypes. Using a combination of high-dimensional flow cytometry and serum proteomics, and unsupervised clustering, three distinct peripheral blood immunological endophenotypes have been recently observed. DMT response was associated with the endophenotype. Patients with endophenotype 3 treated with interferon-beta had higher disease progression and MRI activity compared with treatment with other DMTs (56).

### 2.4 Biomarkers

To date, no clear clinical, imaging, immunologic, or pathologic criteria exist to determine when RRMS transitions into SPMS. There is a need for tools to support early identification of SPMS. The development, validation, and implementation of biomarkers of MS progression are key not only for SPMS diagnosis, but also for monitoring the disease and the long-term treatment outcomes in these patients. To improve SPMS identification and monitoring, the updated definition of SPMS should include clinical evaluations with biomarkers in blood, cerebrospinal fluid (CSF), and imaging techniques. Current MRI assessments in clinical practice only include Gd+ lesions on T1 and new/enlarging lesions on T2, which provide limited information about disease progression. Other pathological processes and potential biomarkers, such as chronic active lesions (CAL; localized areas of compartmentalized inflammation within the CNS that occur in the absence of blood-brain barrier breakdown) and brain and spinal cord atrophy associated with MS progression, specifically with PIRA (57), are currently only being evaluated in research and have not yet been included in routine clinical practice.

Slowly expanding lesions (SELs; contiguous regions of existing T2 lesions showing local expansion) and paramagnetic rim lesions (PRLs; rim of paramagnetic material, mainly iron, that accumulates within activated microglia and macrophages at the edges of the lesion) are promising MRI measures of smoldering inflammation and CAL (58–60). SELs and PRLs are associated with PIRA and with progressive MS (59–65). A higher number, volume or proportion of SELs (66), particularly when combined with PRLs (67), is associated with more pronounced clinical progression than either lesion type alone, suggesting a compounded impact on disease severity when multiple lesion types coexist. While both SELs and PRLs represent CALs, it is unclear how these lesions are interconnected and have different associations with clinical outcomes or SPMS. Together with PRLs and SELs, 18-kDa translocator protein-positive lesions on PET is a promising candidate biomarkers of CAL (68). Further studies are warranted to confirm the role of these biomarkers in SPMS.

Advanced MRI techniques, such as quantitative susceptibility mapping (QSM), diffusion tensor imaging (DTI) and susceptibility-weighted imaging (SWI) are promising for the detection of MS pathogenesis (69–71). Iron content seems to be the primary source of QSM values in deep grey matter and at the edges of rim lesions, with the latter being associated with more severe disability (71). QSM can be acquired with standard field strength (3T) MRI scanners, which makes it a feasible option for clinical settings (72). DTI provides information on the integrity of white matter tracts, which is important in progressive MS where widespread damage occurs beyond visible lesions (73). Recently, the T1-dark rim has been proposed as a novel imaging sign identifiable in a standard 3DT1 gradient-echo inversion-recovery sequence to detect PRLs (74). CALs can be also identified by SWI (60), which makes this technique not only useful for improving MS diagnosis (70) but also for detecting progression.

On the other hand, the rate of retinal layer thinning, measured by optical coherence tomography (OCT), also correlates with disability progression and brain atrophy in MS (75–77). Retinal thinning is associated with PIRA, probably indicating neurodegenerative processes rather than focal inflammation (78). It is worth noting that, although these MRI or OCT features increase the likelihood of accumulating disability, there are still no specific neuroimaging abnormalities that can be considered a hallmark of a distinct MS phenotype, including SPMS.

Several blood and CSF biomarkers have also received great attention in the last years. Neurofilament light chain (NfL), a marker of neuroaxonal injury, and glial fibrillary acidic protein (GFAP), a marker of astrocytic activation, have shown promising results in predicting disease activity and progression (79–85). Elevated levels of serum GFAP (sGFAP) have been associated with accelerated grey matter brain volume loss and disease progression, particularly in nonactive patients (82, 85–87). However, another study revealed that, in the absence of inflammatory activity, changes in serum GFAP did not correlate with disability progression in SPMS patients (88). Elevated baseline levels of NfL in RRMS are predictive of brain and spinal cord atrophy and disability progression (89–91), though GFAP seems to have a stronger correlation with disease progression (85, 92, 93). High NfL levels also correlate with an increased number of relapses and the presence of new or enlarging T2 lesions and T1 Gd+ lesions (89, 94). Some studies have shown that NfL levels are more affected by recent relapses and high-efficacy DMTs (heDMTs) than GFAP levels (92, 93, 95). The persistence of elevated GFAP levels in patients receiving heDMTs might suggest a patient profile with a higher likelihood of disability progression. The combination of high serum NfL and GFAP has been associated with a 4- to 5-fold increased risk of confirmed disability worsening (CDW) and PIRA (86).sNfL and sGFAP could help in differentiating PIRA caused by underlying peripheral inflammation (associated with high sNfL levels) from PIRA associated with smoldering compartmentalized inflammation (observed in cases with elevated sGFAP and low sNfL levels) (96). These findings suggest that NfL and GFAP may provide complementary information about different aspects of the disease progression (96).

Other potential biomarkers of SPMS are soluble triggering receptor expressed on myeloid cells 2 (sTREM2) as a marker of microglial activation, and chitinase-3-like-1 (CHI3L1) as a marker of inflammatory activity (97).

Despite research efforts in identifying and validating these biomarkers of disease progression, their implementation in clinical practice remains limited (**Table 1**). Most of these biomarkers are currently used in research settings only, and their clinical feasibility is still under evaluation. There is a need to establish standardized protocols and cut-off values at an individual level for these biomarkers to be adopted in clinical practice. Current methods of measuring MS disease activity and progression often miss subtle signs of neurodegeneration, such as CAL or brain atrophy. Integrating these “hidden” indicators with biomarkers could offer a more complete picture of disease activity and allow earlier detection of progression (21). First, identification, quantification and monitoring of these biomarkers must be standardized.

**Table 1:** Clinical and paraclinical biomarkers of MS progression.

Biomarkers of conversion to SPMS	Current use in clinical practice	Probability of future use
Clinical biomarkers		
EDSS	High	High
MSFC	High	High
SDMT	High	High
Visual function (contrast, color)	High/medium	High
Imaging biomarkers		
Brain atrophy	Low	Low/medium
SELs	Low	Medium
PRLs	Low	High
Spinal cord atrophy	Low	Low
OCT pRNFL, mGCIPL	High/medium	High/medium
Biomarkers in blood and CSF		
NfL	High/medium	High
GFAP	Low	High/medium
sTREM2	Low	Low
CHI3L1	Low	Low

CHI3L1, chitinase 3-like 1; EDSS, Expanded Disability Status Scale; GFAP, glial fibrillary acidic protein; mGCIPL, macular ganglion cell inner plexiform layer; MSFC, Multiple Sclerosis Functional Composite; NfL, neurofilament light chain; OCT, optical coherence tomography; pRNF, peripapillary retinal nerve fiber layer; PRLs, paramagnetic rim lesions; SDMT, Symbol Digit Modalities Test; SELs, slowly evolving lesions; sTREM2, soluble triggering receptor expressed on myeloid cells 2.

## 3 Difficulty in detecting disease activity in treated patients

### 3.1 Assumption of disease activity

There is a paradox in the use of DMTs indicated for aSPMS. These treatments require patients to present disease activity evidenced by relapses or imaging features of inflammation to initiate treatment (98, 99). However, most patients with RRMS transitioning to SPMS are already treated with DMTs that effectively suppress disease activity (100, 101), making it difficult to detect the required activity to select candidates for initiating the treatments for aSPMS (98). Moreover, MRI has limited utility in detecting new lesions, particularly in cases with a high T2 lesion load, and the use of spinal MRI is not widespread. Theoretically, it could be assumed that if the DMT was discontinued, disease activity would resurge and, in some cases, a rebound effect would be observed. This assumption is based on studies showing an increase in relapses and MRI findings after DMT discontinuation in patients with SPMS (102–105), despite disease activity after discontinuation being lower in SPMS than in RRMS patients (103). A recent study further supports this assumption, demonstrating that patients 50 years and older classified as non-active MS (no evidence of relapse or MRI activity in at least two years, and treated with heDMTs for at least one year) who discontinued the heDMT had an increased probability of inflammatory activity compared with those who continued the heDMT (104).

DMTs indicated for highly active RRMS, such as natalizumab, indeed assume the presence of disease activity. That is, for patients treated with natalizumab who are at higher risk of progressive multifocal leukoencephalopathy (PML), it is recommended to consider their history of disease activity as sufficient criteria to initiate another DMT to reduce the risk of PML (106). Moreover, some European Public Assessment Reports (EPAR) consider patients under DMT as active (107, 108). For example, the EPAR for ofatumumab recognizes that patients currently receiving a DMT for controlling MS inflammatory activity can be considered as fulfilling the ‘activity’ criterion. This is based on the assumption that these patients were experiencing disease activity when the initial DMT was prescribed and that a patient whose inflammatory activity is adequately controlled by a DMT intended to control this activity might not be without activity. The EPAR states that “*the fact that these patients are currently receiving a DMT for controlling the MS inflammatory activity could be considered as a ‘proxy’ of fulfilment of an ‘activity’ criterion*”. Similarly, in the EPAR for ozanimod it is stated that “*switching from other drugs to ozanimod should be allowed despite no clear indication of active disease, for example in patients with side effects or intolerability issues*” (108). Even if an estimation of the impact of patients not meeting the ‘activity’ criterion cannot be directly drawn, it could be inferred that patients switching from their current DMT due to safety or tolerability issues could have a positive risk-benefit ratio (107). Thus, it could be argued that a patient transitioning to SPMS who is treated with a DMT and shows no signs of disease activity should not necessarily be considered as having inactive SPMS (**Figure 1**).

To estimate the likelihood of residual inflammatory activity, the type and duration of treatment, along with other relevant factors (duration of MS, current age, degree of disability, baseline activity before treatment, time without clinical or radiological activity) should be evaluated before concluding that the disease activity observed prior to the initiation of the DMT will remain unchanged, as this activity may have evolved (109). The interruption of a DMT, especially a heDMT, without adequately switching to another DMT, can expose the patient to a higher risk of inflammatory activity (104). Therefore, the neurologist’s judgment, together with the patient preferences, should prevail when a change to a DMT indicated for RMS or aSPMS is required for lack of safety or effectiveness reasons.

The variability in the concept of disease activity is further exemplified by the fact that certain selection criteria for recent clinical trials in nrSPMS, such as in the HERCULES trial, considered only relapses as disease activity, while radiological activity (i.e. gadolinium-enhancing lesions on T1 or new/enlarging T2 lesions) was not taken into account for selecting the patients (25). Thus, patients with disease activity on MRI months or weeks before the study could be included as a nrSPMS patient in the trial (25).

### 3.2 Barriers to activity detection

Detecting disease activity, even when present, is unlikely in treated-RRMS patients who begin to progress, due to several factors. On one hand, less frequent monitoring visits might be conducted in these patients. Some studies have found MRIs are conducted with low frequency in SPMS patients (109, 110). MRI seems to be a more sensitive tool to measuring disease activity than relapses, and therefore, the limited use of MRI in clinical practice might reduce the chance of detecting disease activity in SPMS, as already reported (109). Also, in routine clinical practice, spinal cord MRIs are not regularly performed, and the detection of new/enlarging lesions on T2 is complicated, particularly when there is a high T2 lesion load.

On the other hand, underreporting of relapses by these patients may occur, as they may report minimal symptoms or fail to recognize them as relevant over time (111). Also, physicians may sometimes fail to distinguish relapses from pseudo-relapses, recognize subtle changes, or attribute symptoms to other causes (112), further complicating the detection of activity and progression. Considering this context, we emphasize the importance of face-to-face monitoring in these patients. More frequent monitoring in patients diagnosed with SPMS, compared to the current standard practice, could improve the detection of disease activity. The use of spinal MRI in the follow-up of patients with worsening disability not explained by cranial MRI findings could improve the detection of active lesions, even though it is not currently performed in clinical practice (113). Fluid biomarkers, such as NfL and GFAP, hold promise for establishing progression based on underlying pathological mechanisms.

## 4 Disease-modifying treatments

### 4.1 Approved treatments

The availability of DMTs for SPMS has increased in the last few years, though options remain limited (**Table 2**). More than 25 years ago, IFN-β-1b was the first DMT approved for the treatment of aSPMS, based on positive results in delaying progression in patients with SPMS (114). IFN-β-1a also showed delayed progression in the PRISMS trial (115) and was approved for RMS, even if in the IFN-β-1a trial progression was a secondary endpoint and patients had RMS and a baseline EDSS of 0–5.0. Disappointingly, subsequent clinical trials of these treatments showed conflicting results, with no significant difference in time to confirmed progression between placebo-treated and IFN-β-1b- or IFN-β-1a-treated patients (99, 116–118). *Post-hoc* analyses showed that IFN-β-1a reduced disability progression only when patients had experienced relapses in the two prior years (118, 119). The lack of consistency between studies regarding IFN-β efficacy to delay progression has resulted in limited use of IFN-β to treat SPMS in clinical practice.

**Table 2:** DMTs approved for SPMS and RMS in Europe: pivotal phase III RCT.

DMT; trial	Indication (year of approval)	Selection criteria: age, baseline EDSS (N allocated to treatment; N with SPMS)	Endpoint of disease progression and results
Ponesimod; OPTIMUM (122)	RMS (2021)	18–55 years, EDSS 0 to 5.5 (n=567; SPMS, n=15)	One of the secondary endpoints was time to 12-week CDA. Similar reductions in time to 12-week CDA were observed in the ponesimod group and the teriflunomide group (10.1% vs 12.4%; p=0.29).^1^
Ofatumumab; ASCLEPIOS I, II (121)	RMS (2021)	18–55 years, EDSS 0 to 5.5 (n=946; SPMS, n=27 [ASCLEPIOS I], n=29 [ASCLEPIOS II])	One of the secondary endpoints was proportion of patients with 3-month CDP. The percentage of patients with 3-month CDP was lower with ofatumumab than with teriflunomide (10.9% vs 15.0%; p=0.002).^1^
Siponimod; EXPAND (23)	aSPMS (2020)	18–60 years, EDSS 3.0–6.5 (n=1105; SPMS, all)	The primary endpoint was the time to 3-month CDP. The percentage of patients with 3-month CDP was lower in the siponimod group than in the placebo group (26% vs 32%; p=0·013).
Ocrelizumab; OPERA I, II (120)	RMS (2018)	18–55 years, EDSS 0 to 5.5 (n=821; SPMS, NA)	One of the secondary endpoints was proportion of patients with CDP at 12 weeks. The percentage of patients with CDP at 12 weeks was lower with ocrelizumab than with INFβ-1a (9.1% vs. 13.6%; p<0.001).^1^
Cladribine; CLARITY (123)	RMS (2017)	18–65 years, EDSS 0-6.0 (n=889; SPMS, NA)	One of the secondary endpoints was proportion of patients with 3-month sustained DP. The percentage of patients with 3-month sustained DP was lower with cladribine than placebo, with a 33% reduction in the cladribine 3.5 mg group (p=0.02) and a 31% reduction in the cladribine 5.25 mg group (p=0.03).^1^
IFN-β-1b s.c. European Study Group (114), North American Study Group (116)	aSPMS & RMS (1999)	18–55 years, EDSS 3.0 to 6.5 (European study, n=360; SPMS, all; American study, n=631 [317, 250 µg, 314 160 µg; SPMS, all)	The primary outcome was the time to CP in disability. European study: there was a reduction in the time to CP in patients who received INF-β compared to placebo (p=0.0008; 38.9% in the treated group had CP vs 49.8% in the placebo group). American study: there was no statistically significant difference in the time to CP between groups.
IFN-β-1a s.c.; PRISMS (115)	RMS (1998)	>18 years, EDSS 0–5.0 (n=373; SPMS, n=0)	One of the secondary endpoints was time to sustained progression. Time to sustained progression was longer in both INF-β-1a treatment groups (18.5 months for 22 µg and 21.3 for 44 µg) than in the placebo group (11.9) (p<0·05).^2^

ARR, annualized relapse rate; CDA, confirmed disability accumulation; CP, confirmed progression; CDP, confirmed disability progression; EDSS, Expanded Disability Status Scale; INFβ, Interferon-beta; NA, not available; RCT, randomized clinical trial; RMS, relapsing multiple sclerosis; s.c. subcutaneous; SPMS, secondary progressive multiple sclerosis; ^1^The primary endpoint was the ARR. ^2^The primary endpoint was the relapse count over the course of the study.

Other DMTs, including ocrelizumab, cladribine, ofatumumab, and ponesimod have been approved for RMS. However, these approvals were mainly based on trials that were not specifically designed to assess efficacy in SPMS. These studies included patients with a baseline EDSS ranging from 0 to 5.5 (or 6.0 for cladribine) and used disease activity, measured by the annualized relapse rate, as the primary endpoint (120–123). Disability progression was a secondary outcome in these trials (120–123), and efficacy in SPMS was often inferred from *post-hoc* analyses of small subgroups. The approval of these DMTs, therefore, relied on the assumption that the efficacy in delaying MS progression observed in patients with RRMS and in a small number of patients with SPMS could be generalized to SPMS.

In contrast, the EXPAND study, which led to the approval of siponimod, was specifically designed to evaluate the treatment efficacy in delaying progression in SPMS patients with or without disease activity. The study included patients with a baseline EDSS of 3.0–6.5, and its primary endpoint was time to 3-month confirmed disability progression (CDP) (23). The trial demonstrated a statistically significant 21% relative reduction in the risk of 3-month CDP for patients treated with siponimod compared to those receiving placebo (23). Patients treated with siponimod also had a significantly reduced risk of 6-month CDP, less worsening in processing speed, decreased clinical and radiological disease activity, and a reduction in total brain volume loss (23). *Post-hoc* analysis revealed that siponimod also slowed progression of whole-brain and grey matter atrophy and improved brain tissue integrity/myelination (124). Most of these effects on brain integrity were sustained in the long-term and were more pronounced in patients who initiated siponimod earlier (124). Additionally, in a subgroup analysis of 779 patients with aSPMS, siponimod significantly reduced the risk of disability progression, cognitive decline, and MRI lesions compared to placebo (125).

In the HERCULES trial (25), eligible participants (18–60 years; EDSS: 3.0 - 6.5) were required to have no relapses in the 24 months before screening and disability progression within the 12 months before screening. The primary endpoint was time to onset of 6-month CDP. Tolebrutinib showed a statistically significant 31% risk reduction in time to 6-month CDP compared to placebo. It is worth noting that the study included nrSPMS without clinical relapses for at least 24 months, but the absence of radiological activity was not a selection criterion. Therefore, the nrSPMS population in this study allowed the inclusion of radiologically active and non-active patients.

Masitinib has showed to delayed progression (primary endpoint: EDSS change from baseline) in PPMS and nrSPMS in a phase 3 trial; a confirmatory phase 3 study is ongoing and will provide further data (126). Another ongoing study assesses the efficacy and safety of fenebrutinib compared with ocrelizumab in PPMS in disability progression (primary endpoint: time to onset of composite 12-week CDP) (127). Other clinical trials assessing the efficacy of treatments on disability progression as primary endpoint in patients with SPMS have failed to achieve positive results (128), highlighting the difficulties in developing effective treatments for SPMS. Multiple factors should be considered to improve the design of these trials (128). First, the profile of included patients should align with the mechanism of action of the drug (i.e. immunomodulation, neuroprotection, or remyelination). For example, an immunomodulation mechanism that targets inflammation would be relevant for patients with aSPMS. In contrast, for patients with nrSPMS, the treatment should target remyelination or neuroprotection rather than inflammation. Also, including a more heterogeneous profile, such as older adults and those with comorbidities, would increase the generalization of results. Second, the primary endpoint should be a sensitive measure that captures several aspects of progression, such as the EDSS-plus or other clinically meaningful composite measures; relevant biomarkers of progression and patient-reported outcomes (PROs) should also be included as secondary outcomes. Lastly, trials could be more efficient with a multi-arm design, where several active drugs are compared to the placebo arm, or a multi-stage design that allows modifying the study protocol based on interim results (128).

### 4.2 Discontinuation

The question of whether, and if yes, when and how, to safely discontinue DMTs in older and stable SPMS patients remains a topic of ongoing debate. Research on DMT discontinuation has yielded contradictory results about relapse rates and progression, probably due to differences in disease activity before discontinuation, age at discontinuation and treatment efficacy (129–132).

One of the most anticipated results was the findings of the DISCOMS trial (130). This trial allocated stable patients 55 years or older to either stop or to continue their DMT and had a 2-year follow-up. Those who discontinued experienced an increase in clinical or radiological disease activity (12.2%) compared with those who continued DMTs (4.7%), with no significant differences in increased disability. With an absolute difference of 7.5%, the authors were unable to reject the null hypothesis that DMT discontinuation was non-inferior to continuation. The study included a small proportion of patients treated with heDMTs (9%), and therefore, the conclusion of this trial cannot be generalized to all DMTs.

The STOP-I-SEP (NCT03653273) will provide further evidence on the impact of discontinuation on MS. The study assesses the effect of treatment discontinuation in disability progression, disease activity and health-related quality of life (HRQoL) in SPMS patients older than 50 years. The DOT-MS study (NCT04260711), which evaluated disease activity, disability and PROs in patients treated with moderate-efficacy DMTs (meDMTs; interferons, glatiramer acetate, dimethyl fumarate, or teriflunomide) was terminated early after an interim analysis revealed increased disease activity above the predefined limit in the group that discontinued treatment (133).

While we await these results, real-world evidence provides valuable data. A recent meta-analysis, which included 22 real-world studies and 2942 patients followed for 1–7 years after discontinuation, found that the risk of relapses after DMT discontinuation became negligible (i.e. < 1% per year) around the age of 60, and after either 10 years of DMT use or 8 years of stable disease (129). However, as in the DISCOMS trial, the majority of patients included in the study were treated with meDMTs. This raises the question of whether the impact of discontinuation on disease activity might depend on the efficacy of the treatments (heDMTs vs. meDMTs). A recent study using propensity-matching scores evaluated disease activity in patients 50 years and older with nonactive MS who either continued or discontinued heDMT (rituximab, ocrelizumab, natalizumab, and fingolimod). The study found that the risk of relapse was significantly higher in patients who discontinued the heDMT compared with patients who continued but varied greatly according to the DMT (104).

The integration of findings from clinical trials and real-world studies can guide clinicians in making informed decisions regarding DMT discontinuation. However, to date, there is a lack of studies specifically evaluating the discontinuation of DMTs approved for SPMS, and the long-term effects and risks of stopping these therapies are not well understood yet. When considering discontinuing treatment in a patient with SPMS, several factors must be considered, such as age, duration of MS, accumulated disability, progression rate, time of clinical/radiological stability, DMT type and safety, and comorbidities. The risk-benefit balance of discontinuing a DMT should be carefully addressed for each individual patient.

## 5 Discussing the progressive phase with patients

Discussing the progressive phase of MS with patients is a delicate aspect. Most patients want to know their long-term prognosis as soon as possible (134). However, predicting disease progression is difficult, as the variability in the rate of progression is high, although several prognostic factors influencing long-term progression have been identified and can help to anticipate disability (135). Also, new tools such as machine learning can be used to predict conversion to a secondary progressive course, confirmed disability accumulation, and disease severity with rapid accumulation of disability (136, 137).

There is no consensus on the best time to introduce discussions with patients about MS progression. Some neurologists prefer to address the issue early to facilitate informed decision-making and planning. In contrast, others delay the conversation out of concern that the conversation may increase anxiety and delay decisions regarding treatment (138). One of the main difficulties in starting these discussions has been the lack of effective treatments in reducing disease progression. However, in the last few years, the approval of DMTs for aSPMS has provided an option to treat these patients (23, 124, 125). The positive results from clinical trials of other treatments in delaying progression (25, 126) give hope that additional DMTs that modify the disease course and delay progression will be available.

Something important to consider when addressing the progressive phase with patients is the fact that most patients have probably already come across the concept of disease progression when searching for information online about MS (139). Patients can now easily access medical and scientific information by using artificial intelligence (AI)-powered large language models. These tools have transformed information retrieval, as they provide instant, context-specific explanations of medical concepts in a human-like manner (140). Furthermore, online patient communities and social media platforms create spaces where patients can share their experiences (141). As a result, physician–patient communication must evolve to match the increasing accessibility of medical knowledge.

Neurologists should be prepared to engage with patients and caregivers in discussions that clarify, validate, and contextualize the information gathered from AI tools and digital platforms. Effective communication—characterized by empathy, validation of emotions, clarity, and active listening—plays a key role in managing patients. A recent study evaluated the preferences of patients with MS towards responses to frequently asked health-related questions provided by either neurologists or by ChatGPT. Patients, who were unaware of who generated the response, perceived ChatGPT responses as more empathetic compared to responses from neurologists, although patients with higher levels of education showed lower satisfaction towards the responses created by ChatGPT (142). In fact, empathy has been reported by older patients with MS as an area for improvement among neurologists (143). Therefore, improving soft skills, especially oral communication skills, is more important than ever when discussing sensitive topics with patients, such as disease progression. Including the family in the conversation and involving the patient in shared decision-making of DMT and symptomatic treatment are also relevant for increasing treatment satisfaction (144, 145).

A key strategy in discussing progression is having an ongoing conversation rather than a one-time disclosure. Instead of presenting progression as an inevitable outcome, neurologists could emphasize that progression is a gradual process, often subtle and that proactive management—including lifestyle modifications, symptomatic treatment, and rehabilitation strategies—can improve long-term outcomes. Patients’ emotional responses to information about MS are shaped by the way their providers communicate prognosis and treatment expectations. One of the most important factors influencing patients’ satisfaction with the MS diagnosis is adequate emotional support (146). By acknowledging patient concerns while providing reassurance that their care plan is adaptable, neurologists can help patients engage with their treatment strategy without feeling a sense of impending loss.

Discussing MS progression requires a patient-centered, stepwise approach that evolves over time. While the initial focus should be on controlling disease activity and maximizing HRQoL, patients should also be prepared for the possibility of progression. When discussing progression, neurologists should also clarify that disease progression is not only defined by motor decline but can include cognitive, sensory, and functional changes, which have an impact on HRQoL (147), even in the absence of relapses or MRI lesions (148).

Patients often notice these subtle changes before they are clinically confirmed, and thus, neurologists must listen carefully and integrate these patient-reported symptoms into their assessments. There is no standard guideline on when to address progression, and therefore, neurologists should tailor these discussions to individual patients, considering their level of health literacy, emotional readiness, and personal concerns.

## 6 Concluding remarks

Optimizing the management of SPMS requires a comprehensive approach. First, there is a critical need to establish a unified definition of SPMS that captures the early stages of disease progression, where the introduction of DMTs may be most beneficial. This definition should incorporate an understanding of the underlying pathological processes and be supported by biomarkers that can be routinely applied in clinical practice.

Given that neurodegenerative changes begin early in the disease course and contribute to long-term disability, these processes should be identified as soon as possible. Current clinical measures alone may not fully capture disease activity and the underlying neurodegeneration that may be masked by compensatory mechanisms during the early stages of MS. Emerging biomarkers, such as SELs and GFAP, hold promise for detecting early neurodegenerative changes in the near future.

Furthermore, the initiation of DMTs with proven efficacy in reducing progression should be used as soon as the patient transitions to SPMS. The difficulties in identifying SPMS in patients currently treated with DMTs that reduce disease activity should not prevent the decision to start DMTs indicated for aSPMS. The use of current DMTs could be considered an assumption of ongoing clinical activity.

To support evidence-based decisions in MS management, clinical trials for MS treatments should include more patients with SPMS. Also, discussing MS progression with patients requires a patient-centered, stepwise approach, balancing transparency, emotional support, and shared decision-making. By addressing these issues, we can improve long-term outcomes for patients with SPMS and provide more effective, safe and personalized care.
